# A prospective study of bowel preparation for colonoscopy with polyethylene glycol-electrolyte solution versus sodium phosphate in Lynch syndrome: a randomized trial

**DOI:** 10.1007/s10689-012-9517-7

**Published:** 2012-02-23

**Authors:** Maria W. J. van Vugt van Pinxteren, Mariëtte C. A. van Kouwen, Martijn G. H. van Oijen, Theo van Achterberg, Fokko M. Nagengast

**Affiliations:** 1 Department of Gastroenterology and Hepatology, Radboud University Nijmegen Medical Centre, P.O.Box 9101, 6500 HB Nijmegen, The Netherlands; 2 Department of Gastroenterology and Hepatology, University Medical Centre Utrecht, P.O.Box 85500, 3508 GA Utrecht, The Netherlands; 3 Scientific Institute for Quality of Healthcare, Radboud University Nijmegen Medical Centre, Nijmegen, P.O.Box 9101, 6500 HB Nijmegen, The Netherlands

**Keywords:** Lynch syndrome, Colonoscopy, Polyethylene glycol, Sodium phosphate

## Abstract

Lynch gene carriers undergo regular surveillance colonoscopies. Polyethylene glycol-electrolyte solution (PEG) is routinely prescribed for bowel cleansing, but often poorly tolerated by patients. Sodium phosphate (NaP) may be an alternative. Prospective and random comparison of bowel preparation with PEG and NaP on colon cleansing and patients’ acceptance. Patients, who previously underwent a colonoscopy, were invited to participate and randomly assigned to either PEG or NaP. They were asked to fill in a questionnaire about preparation tolerability and future preferences. The endoscopist filled out a report about the quality of colon cleansing. 125 Patients were included in the study. Nine (7%) were excluded because of missing data. The remaining 116 patients (53 PEG and 63 NaP) were included in the analysis. Baseline characteristics did not differ between groups. Before colonoscopy 20 (38%) patients using PEG experienced the preparation almost intolerable, in contrast to 7(11%) of those using NaP (*P* = 0.001). Eleven patients in the PEG group and 48 in the NaP group would prefer NaP in the future. The colonoscopy was poorly tolerated in 17% of the individuals in both groups (*P* = 0.963). The endoscopist observed a more than 75% clean colon in 83% of patients on PEG and in 71% of patients on NaP (*P* = 0.076), however the coecum (*P* = 0.025) and ascending colon was cleaner after PEG. Lynch patients tolerated NaP better and preferred this formula for future bowel preparation. Colon cleansing was suboptimal with both treatments with a tendency towards a cleaner proximal colon with PEG.

## Introduction

Up to 5% of all colorectal cancer cases are attributed to the Lynch syndrome [1].Lynch syndrome is caused by germ line mutations in one of the mismatch repair (MMR) genes MLH1, MSH2, MSH6 or PMS2 [2]. Lynch syndrome gene carriers are recommended to undergo regular endoscopic surveillance of their colon. This surveillance is preferably carried out by colonoscopy, [3, 4] in order to detect adenomas or less frequently early stages of colon cancer. It is important that the complete colonic mucosa can be inspected, especially the proximal colon, since most tumors in Lynch syndrome develop in this part of the colon. For inspection, a meticulously clean colon is therefore a prerequisite. Various colon-cleansing solutions have been studied in the past. Of these, a polyethylene glycol-electrolyte solution (PEG) is routinely prescribed for bowel cleansing, but it is often poorly tolerated [5–8]. Sodium phosphate (NaP) may be an effective alternative, and is often better tolerated because of the small amount of liquid intake. However, it has other disadvantages like acute phosphate nephropathy and should be used with caution by at risk individuals and not be prescribed to patients with cardial and/or renal failure [3, 5, 7–12]. Earlier studies with PEG and NaP have shown an excellent clean colon in 18–80% [7–12]. Patients mentioned equal acceptability about the bowel cleansing. The tolerability of bowel cleansing was about 60% [3, 6, 8–10, 12–14]. In these earlier studies it was often not clear if the study participants underwent a surveillance colonoscopy previously, nor was information about special patient groups available. The aim of this study was to randomly compare the effects of preparation on bowel cleansing with PEG or NaP and to evaluate the acceptance of the two solutions by Lynch syndrome gene carriers who used PEG as colon cleansing in the past.

## Methods

### Patients

This single blinded study was carried out at the department of Gastroenterology and Hepatology in a Dutch University hospital. During 1 year, Lynch syndrome gene carriers, who were scheduled for a surveillance colonoscopy, were asked to participate in the study. They were enrolled in a surveillance colonoscopy program and underwent at least one colonoscopy previously at which they used PEG as preparation on bowel cleansing. Patients with a history of colonic surgery were excluded. All procedures took place in the afternoon. Participants were randomly and single-blindly assigned to either PEG (Norgine bv, Amsterdam, the Netherlands) or NaP (Ferring, Hoofddorp, the Netherlands).

Lynch syndrome gene carriers are generally healthy persons without physical and medical restrictions to use NaP.

Patients who were assigned to the PEG-group were given a special dietary prescription. 2 days before colonoscopy only lightly digestible products will be consumed and at the day before colonoscopy only fluid products. In addition, at the day before colonoscopy they had to use 15 gram magnesium sulfate and 10 mg bisacodyl (both orally) (Boehringer Ingelheim, Alkmaar, the Netherlands). At the day of colonoscopy patients had to use 4 litres PEG or at least enough until the stool is clear. PEG was not given as a split-dose. Patients who were assigned to the NaP group used a lightly digestible breakfast and lunch at the day before colonoscopy, as prescribed in the instructions for use. After lunch at the day before colonoscopy, they consumed only clear fluids, at least 3 litres until the colonoscopy. In the evening before colonoscopy 45 ml of NaP had to be taken. Three hours before colonoscopy another 45 ml of NaP had to be used. Informed consent was obtained and the study was approved by the local Medical Ethical Committee.

### Instruments

The quality of bowel cleansing was assessed for each segment of the colon (descending, transverse and ascending colon and the coecum) and graded as excellent (no fecal matter), good (small amounts of thin, liquid fecal matter; easy to remove), fair (moderate amounts of thick liquid fecal matter; difficult to remove) and poor (large amounts of thick liquid or solid fecal matter; not to remove) (Table 1).

**Table 1:** Cleansing grading score by the endoscopist

Excellent	No fecal matter in the colon
Good	Small amounts of thin, liquid fecal matter in the colon; easy to remove
Fair	Moderate amounts of thick liquid fecal matter in the colon; difficult to remove
Poor	Large amounts of thick liquid or solid fecal matter in the colon; unable to remove

During the day of preparation before colonoscopy and 1 week after colonoscopy, patients were asked to fill in a questionnaire about their experiences with the dietary pattern, potential interference with daily activities, tolerance and side effects of the bowel preparation and the taste of the liquid. They were also asked to mention their preferences for a bowel preparation in the future. The questionnaires were filled in at home before and 1 week after colonoscopy. The endoscopist (blinded for the way of bowel cleansing) filled out a report about the effectiveness of bowel cleansing and the duration of the introduction time and the time of the whole colonoscopy ranked as 12.5 min or less, 12.5–25 min, 25–37.5 min and more than 37.5 min. The endoscopy nurse (also blinded for the way of bowel cleansing) filled out a report about the observation of signs of pain during colonoscopy. The pain score was ranked on a visual analog score from 1 to 5 as no pain (1); mild pain (2); moderate pain (3); severe pain and more sedation is necessary (4) and interrupted colonoscopy because of very severe pain (5).

### Statistical analysis

Data were collected and entered into an electronic database. All calculations were carried out using the Statistical Package for the Social Sciences Program (SPSS version 16.0 (SPSS, Chicago, Illinois, USA). Frequency tables were provided for description of patients and bowel cleansing characteristics and were compared between groups. Descriptive statistics were computed for all variables. These included means, medians and standard deviations. Pearson’s Chi-square test was used to analyze associations between baseline characteristics, differences for PEG and NaP, quality and side effects of bowel cleansing and results of cleansing. *P* value below 0.05 (two-sided) was considered statistically significant.

## Results

During a 1 year study period, 125 consecutive Lynch gene carriers participated in the study (100%). Of these 125 patients, nine (7%) were excluded because of missing data; they did not send back one or both questionnaires. The remaining 116 patients (M/F 58/58, mean age 50 ± 30 years) were included in the analysis. Fifty-three patients received PEG and 63 NaP. Twenty-three (20%) participants underwent one colonoscopy in the past, 59 (51%) underwent 2-5 colonoscopies, 31 (27%) had 6-10 colonoscopies and 3 (2%) patients underwent more than 10 colonoscopies (median 3).

**Fig. 1:**
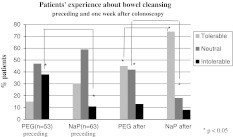
Patients’experience about bowel cleansing

In only one patient the special diet at the day before colonoscopy interfered with daily activities (0.6%). Eighty four percent of all patients did not mention any or only little problems at the day before the colonoscopy. At the day of colonoscopy the preparation with the laxative interfered with their daily activities in nineteen patients (16%). Twenty patients using PEG (38%) found the preparation almost intolerable in contrast to seven (11%) patients using NaP (*P* = 0.001) before colonoscopy. A week after colonoscopy 24 (45%) patients using PEG and 47 (75%) of those using NaP evaluated the preparation as tolerable (*P* = 0.001). Twenty-two (42%) patients using PEG and 11 (17.5%) using NaP mentioned the preparation as neutral (*P* = 0.004). At least seven (13%) patients using PEG (four of them evaluated the preparation as more difficult afterwards) and five (8%) patients using NaP) (the same five patients who communicated this before) evaluated the preparation as intolerable (Fig. 1). The most commonly mentioned side effects of the preparation were nausea, abdominal cramps and flatulence; these side effects were mentioned in the instructions of PEG and NaP. Patients also commonly mentioned that they suffered from feeling cold; this was not mentioned as a possible side effect in the instructions of PEG and NaP (Table 2). Sixty percent of all patients using PEG and 58% of patients using NaP used 4 l of fluids or more before colonoscopy; equal to or more than prescribed.

**Table 2:** Side effects of bowel preparation (measured before colonoscopy)

	PEG-group (*n*, %)	NaP-group (*n*, %)	*P* value
Nausea	24 (21)	20 (17)	0.134
Vomiting	4 (3)	6 (5)	0.706
Abdominal cramps	23 (20)	27 (23)	0.953
Flatulence	15 (13)	13 (11)	0.336
Physical cooling	34 (30)	34 (30)	0.267
Insomnia	8 (7)	8 (7)	0.709

Twenty-one patients (18%) had a preference for using PEG in the future (six of them randomized to NaP in this study), 59 (51%) preferred to use NaP (11 of them randomized to PEG in this study) and 31 (27%) of the patients had no preference (22 PEG/9 NaP). Five patients (4%) did not mention their preference. In nine (17%) of the individuals using PEG and in 11 (18%) of the NaP participants the colonoscopy was equally poorly tolerated.

The endoscopist (*n* = 8) reported a clean colon in 44 participants (83%) using PEG and also in 44 patients (71%) using NaP. In 19 (36%) of the total group of PEG users and 13 (21%) of the NaP users the endoscopist observed a more than 75% complete clean colon (*P* = 0.076). In 42% of the PEG group the coecum was excellent clean, compared to 22% in the NaP group (*P* = 0.02) (Table 3). In 11% the ascending colon was poorly cleaned in the NaP group, compared to 2% in the PEG group (*P* = 0.05). The introduction time into the coecum and the duration of the whole colonoscopy did not significant differ between both groups. In both PEG (29%) and NaP (25%) the introduction time into the coecum was about 12.5 min (*P* = 0.645) while in PEG (9%) and NaP (13%) the introduction time has taken more than 37.5 min. The majority of patients (80%) received midazolam as sedation during the colonoscopy. During the colonoscopy the endoscopy nurse observed pain on a visual analog score in 73 (33 PEG and 40 NaP) of all patients (47%); of them 17 (15%) suffered from a lot of pain (6 PEG and 11 NaP). In one case (NaP) the colonoscopy was stopped because of extreme pain. Fifty-five patients mentioned complaints (flatulence or abdominal pain) after colonoscopy, which always disappeared within 24 h.

**Table 3:** Quality of bowel cleansing for segments of the colon (*n*, %)

	Coecum		Ascending colon		Transverse colon		Descending colon	
	PEG	NaP	PEG	NaP	PEG	NaP	PEG	NaP
Excellent	22(42)^#^	14(22)	19(36)	14(22)	17(32)	12(19)	14(26)	12(19)
Good	16(30)	20(32)	16(30)	26(41)	24(45)	32(51)	25(47)	25(40)
Fair	12(22)	19(30)	17(32)	16(26)	11(21)	18(29)	12(23)	24(38)
Poor	3(6)	10(16)	1(2)^#^	7(11)	1(2)	1(1)	2(4)	2(3)

^#^ *P* = < 0.05 in favour of PEG

## Discussion

Persons at risk for colon cancer, especially Lynch syndrome gene carriers, benefit from regular surveillance colonoscopies [15]. For an optimal detection of colonic neoplasia a clean colon is very important [11]. To achieve a clean colon, compliance with the bowel preparation regimen is necessary. The most commonly used bowel cleansing solutions are often burdensome to patients because of the large amounts of fluids and their bad taste [11]. To our knowledge no information is available about the experience of bowel preparation in Lynch patients in a regular colonoscopy program. In this study all patients were able to tolerate the preparation regime, in contrast to some other studies [9, 13].

### Clean colon

In patients with Lynch syndrome the increased risk for developing colon cancer is highest in the colon proximal to the splenic flexure [16]. It is therefore particularly important to examine this part meticulously and it is essential that this part of the colon is excellently cleaned. We found a comparable clean colon in the PEG and NaP group [17]. In assessing this, we used the same criteria for “poor” clean, “fair” clean, “good” clean and “excellent” clean colon as described in previous studies [5, 7, 8, 11–14, 18]. Also the same segments of the colon were evaluated [7, 9, 11, 12, 18]. It was remarkable that, in contrast to other studies, we found an excellent clean colon only in 27.6% of the participants, regardless of the bowel cleansing. A significant difference in an excellent clean coecum was found in favour for PEG preparation (42% vs. 22%). Moreover, in the ascending colon the quality of bowel cleansing was significantly more poor in the NaP group (11% vs. 2%). The cleansing efficacy of PEG in non-Lynch patients was comparable for both left and transverse colon, but the cleanliness was also superior in the coecum and ascending colon in a previous Dutch study [9].

### Tolerance

Nausea was mentioned as a moderate side effect of the colon cleansing in both PEG and NaP groups; we found no difference between the groups, which was in accordance with other studies [5, 7, 8, 11, 14].

In our study both PEG and NaP users often suffered from feeling cold; in other studies no information was found about this side effect. The 60% of patients, who drank 4 litre fluids or more, did not consume the drinks at the same time. Patients who used PEG, drank this amount at the day of colonoscopy and patients’ who used NaP, drank this amount in 2 days; nevertheless many of them mentioned this side effect.

Twenty-five percent of all patients poorly tolerated the cleansing preparation; however, a week after the colonoscopy only 10% of the participants mentioned that the preparation was not tolerable. Probably not only the taste and the amount of the cleansing liquid are of influence on patients’ perception. It is also possible that fear for the colonoscopy itself, the fear for the outcome of the colonoscopy and the consequences of these results to the future influenced patients’ perception.

Overall, in contrast to some other studies, [12, 14] because of the amount of the liquid, the acceptance of the bowel preparation was in favour for NaP, however the taste of the liquid was similar for both groups [9].

### Pain

The information of the endoscopy nurse about the observation of pain during the endoscopy was in line with the patients’ information about their experience of the colonoscopy. In the PEG group, there was a tendency towards less pain.

### Preference

Patients who used PEG or NaP most of time prefer to use the same formula for bowel cleansing in the future or they mentioned no preference. In the total group 51% was in favour of NaP. The same acceptability was found in other studies [11, 14]. Thirty-three percent of the users in the PEG group, who did not endure PEG in the present study, mentioned a preference for NaP, even although they did not know anything about the liquid’s taste or possible side effects of NaP.

In our study PEG and NaP are both effective as preparation for bowel cleansing with a tendency to a cleaner colon with PEG. This is in accordance with some earlier studies, [5, 6, 8, 9, 11, 12, 14, 19, 20] but the most important observation was a significantly more clean proximal part of the colon with PEG preparation [21]. This last finding is important with regards to the afore-mentioned high risk of neoplasia in the proximal colon in Lynch syndrome.

To our knowledge this is the first study relating to bowel cleansing in Lynch gene carriers. Although this study was conducted in a small group of gene carriers, who visited the outpatient clinic in one hospital, we conclude that bowel preparation, in this representive group of Lynch gene carriers, is not optimal with both preparations. In the proximal part of the colon PEG seems to clean the mucosa better than NaP. These findings suggest that for optimal (proximal) bowel cleansing in Lynch syndrome PEG is a better option than NaP. The efficacy of bowel cleansing should always be more important than patient preferences in determining the choice of bowel preparation. Maybe other reasons than taste and amount of cleansing liquid are of influence at patients’ acceptability for bowel cleansing. So it seems mandatory that in patients at high risk of (proximal) colon cancer, better cleaning regimes as well as patients’ acceptance should be developed and investigated.

## Abbreviations

PEG: Polyethylene glycol-electrolyte solution
NaP: Sodium phosphate
MMR: Mismatch repair
